# A novel detection methodology for HER2 protein quantitation in formalin-fixed, paraffin embedded clinical samples using fluorescent nanoparticles: an analytical and clinical validation study

**DOI:** 10.1186/s12885-018-5172-1

**Published:** 2018-12-18

**Authors:** David G. Hicks, Brandon Buscaglia, Hideki Goda, Loralee McMahon, Takako Natori, Bradley Turner, Armen Soukiazian, Hisatake Okada, Yasushi Nakano

**Affiliations:** 1 Department of Pathology and Laboratory Medicine, University of Rochester Medical Center, Rochester, NY USA; 2 Konica Minolta INC., Bio Health Care Business Development Division, Corporate R&D Headquarters, No. 1 Sakura-machi, Hino-shi Tokyo, 191-8511 Japan

**Keywords:** Breast cancer, HER2, Quantitative protein analysis, Immunohistochemistry, In situ hybridization, Phosphor integrated dot fluorescent nanoparticles, Neoadjuvant trastuzumab based chemotherapy

## Abstract

**Background:**

Clinical assays for the assessment of the human epidermal growth factor receptor-2 (HER2) status in breast cancer include immunohistochemistry (IHC) and in situ hybridization (ISH), both of which have limitations. Recent studies have suggested that a more quantitative approach to the measurement of HER2 protein expression may improve specificity in selecting patients for HER-2 targeted therapy. In the current study, we have used HER2 expression in breast cancer cell lines and clinical samples as a model to explore the potential utility of a novel immunodetection technique, using streptavidin coated Phosphor Integrated Dot fluorescent nanoparticles (PID), which can be quantitatively measured using computer analysis.

**Methods:**

The expression of HER2 protein in cell lines was evaluated with antibody-binding capacity using fluorescence-activated cell sorting (FACS) for comparison with PID measurements to test for correlations with existing quantitative protein analysis methodologies. Various other analytic validation tests were also performed, including accuracy, precision, sensitivity, robustness and reproducibility. A methods comparison study investigated correlations between PID versus IHC and ISH in clinical samples. Lastly, we measured HER2 protein expression using PID in the pretreatment biopsies from 34 HER2-positive carcinomas that had undergone neoadjuvant trastuzumab-based chemotherapy.

**Results:**

In the analytic validation, PID HER2 measurements showed a strong linear correlation with FACS analysis in breast cell lines, and demonstrated significant correlations with all aspects of precision, sensitivity, robustness and reproducibility. PID also showed strong correlations with conventional HER2 testing methodologies (IHC and ISH). In the neoadjuvant study, patients with a pathologic complete response (pCR) had a significantly higher PID score compared with patients who did not achieve a pCR (*p* = 0.011), and was significantly correlated to residual cancer burden (RCB) class (*p* = 0.026, R^2^ = 0.9975).

**Conclusions:**

Analytic testing of PID showed that it may be a viable testing methodology that could offer advantages over other experimental or conventional biomarker diagnostic methodologies. Our data also suggests that PID quantitation of HER2 protein may offer an improvement over conventional HER2 testing in the selection of patients who will be the most likely to benefit from HER2-targeted therapy. Further studies with a larger cohort are warranted.

## Background

The human epidermal growth factor receptor-2 (HER2) is a transmembrane tyrosine kinase receptor that plays an important role in regulating normal cell growth, differentiation and survival [1]. Over-expression of the ERBB2 gene or HER2 protein occurs in 15–20% of all invasive breast cancers and has an important bearing on prognosis, as majority of HER2-positive tumors are associated with a more aggressive clinical course and typically a poor outcome [2]. Fortunately, new targeted therapies can directly inhibit biomarker receptors including the drug trastuzumab, which is a humanized monoclonal antibody that directly targets HER2 over-expression by binding with high affinity to an extracellular epitope of the HER2 receptor. Targeting HER2 over-expression with trastuzumab in breast cancer has proven to be remarkably successful in clinical trials, which have demonstrated significant improvements in disease free survival and overall survival in breast cancer patients in the metastatic setting [3] and adjuvant setting [4, 5]. More recently, targeting HER2 over-expression has demonstrated an excellent pathologic response to therapy in the neoadjuvant setting [6, 7]. Clinical assays to assess the HER2 status in breast cancer patients that are being considered for targeted therapy include immunohistochemistry (IHC), which detects protein over-expression, and in situ hybridization (ISH), which detects gene amplification [8]. Both assays have been clinically validated for predicting which patients will benefit from treatment that targets HER2 over-expression [9, 10], and both assays have received FDA approval. With the development of new HER2-targeting drugs and the expanding options for targeting the HER2 pathway in breast cancer and other solid tumors [11–14], accurate and reliable HER2 testing to ensure that the right patients receive the right treatment is critical.

Both the IHC and ISH methodologies have limitations. Among “HER2-positive” tumors defined by consensus criterion [9, 15] there is a range of variability of results in terms of HER2 gene amplification and the semi-quantitative measurement of protein over-expression by the conventional ISH and IHC methods [16]. IHC utilizes chromogenic detection of the receptor protein and has a limited dynamic range where the test is linear, limiting the ability to obtain accurate quantitative results [17], which may be clinically useful. HER2 ISH analysis represents a surrogate for the over-expression of the HER2 receptor protein, and is more quantitative than IHC, utilizing fluorescent dyes to enumerate gene copy numbers; however, these fluorescent signals photo bleach and fade over time [18]. Given that the target of the drug trastuzumab is the HER2 receptor protein, novel detection systems that accurately and quantitatively detect HER2 protein on the membrane of tumor cells in formalin fixed, paraffin embedded (FFPE) clinical samples in a linear fashion over a broad dynamic range would be advantageous, and may provide clinically useful information. A number of new methodologies have been developed to obtain more quantitative HER2 results from FFPE breast cancer samples at the protein level, including AQUA technology [19] and HERmark [20, 21]. Both of these technologies have demonstrated promising clinical utility; however, they require special equipment and are currently only offered at central reference laboratories, limiting broader clinical applications. Various other methods, including enzyme-linked immunosorbent assay, fluorescence-activated cell sorting (FACS), and matrix-assisted laser desorption/ionization, have all been developed to quantify protein expression; however, these methods cannot simultaneously evaluate cellular morphology [22–25].

**Fig. 1:**
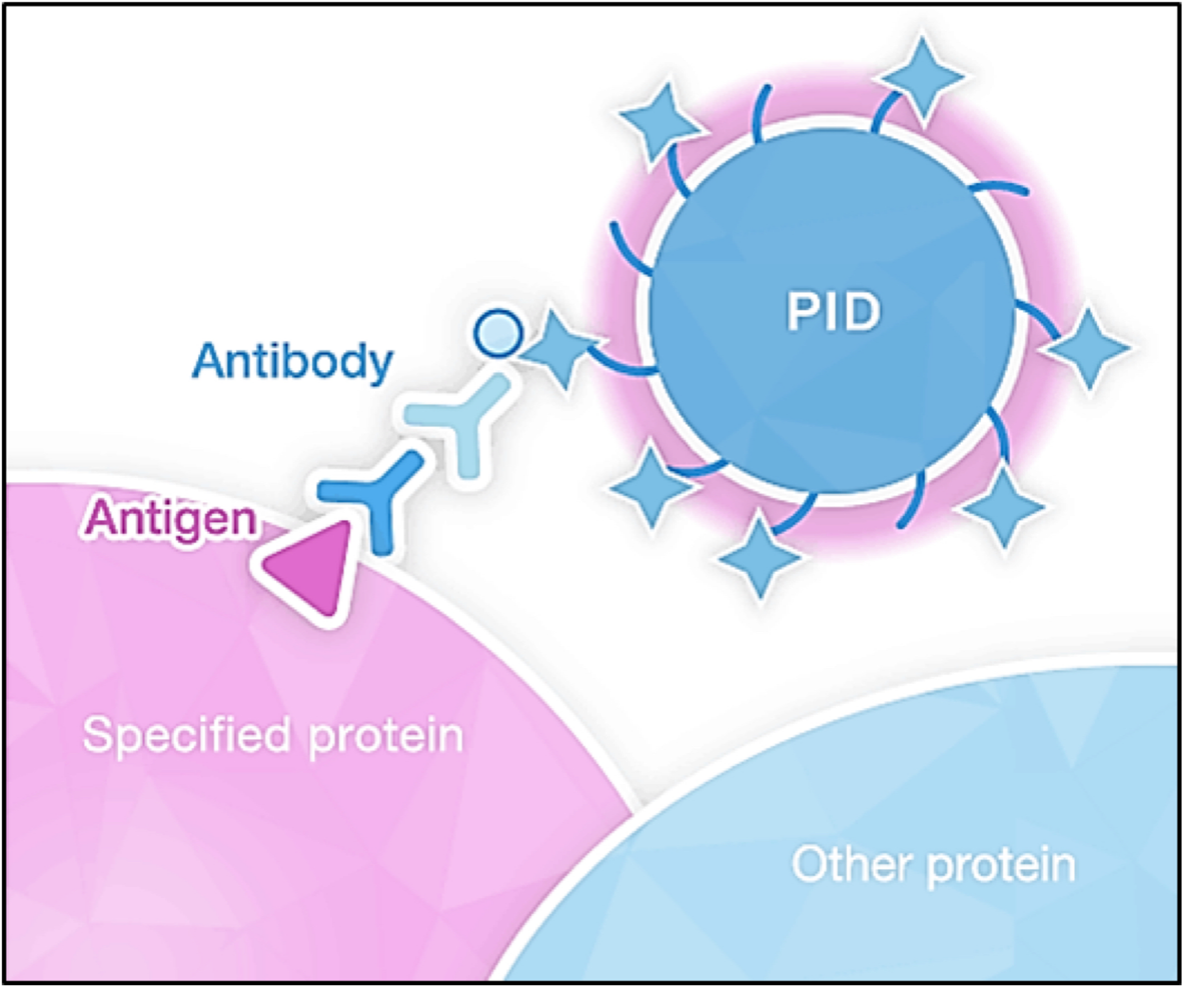
Structure of PID nanoparticles and binding method with antigen. PID nanoparticles consist of approximately 100,000 perylene diimide, and its surface was coated with streptavidin via PEG chains. The staining method using PID is in principle similar to classic immunohistochemistry (IHC). PID nanoparticles bind to antigen on the specific protein via primary antibody and biotin labeled secondary antibody. These nano-particles are 10,000 times brighter compared to conventional organic dyes, with high photostability

**Fig. 2:**
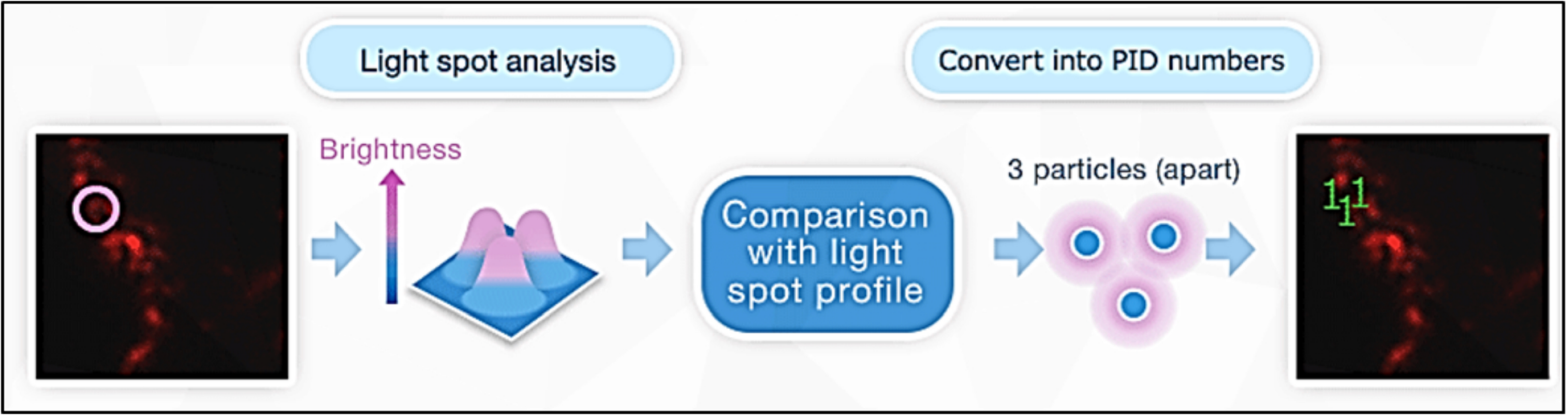
Schematic to measure the number of PID nanoparticles on the tissue surface. The number of PID nanoparticles on the digital image is calculated based on the light spot profile. This process consists of defining the area of a bright spot derived from PID nanoparticles using high-pass-filtered images and estimating the number of PID nanoparticles in the bright spot by using the data and correlating the number of particles detected by observed SEM and fluorescence intensity

Streptavidin-coated phosphor integrated dot fluorescent nanoparticles (PID) are brightly fluorescent particles of controlled diameter that are 100 times brighter compared with commercially available Q-dots and 10,000 time brighter compared with conventional organic dyes [26] (Fig. 1). PID nanoparticles show high photostability, do not photo bleach or fade over time, and can be used as a novel immunofluorescence detection system to quantitatively measure proteins in FFPE tissue sections (Figs. 1 and 2). An image-processing method can be used to calculate the number of PID nanoparticles on images acquired by using a versatile optical system, with a read out that includes the average PID/cell (PID score/cells) and the average number of PID nanoparticles per unit area (100μm^2^) (PID score/ROI100μm^2^) [26]. Gonda et al., previously conducted an analytic and clinical validation study for the potential utility of PID particles in quantitating HER2 membrane protein in well characterized breast cancer cell lines that demonstrated a range of levels of HER2 expression. Briefly, their results showed significant analytic performance in parameters that included precision, repeatability, linearity, dynamic range and sensitivity [26]. In the current study, additional analytic validation of PID testing in six breast cancer cell lines was performed to provide further evidence and support for PID performance. Compatibility of PID testing with clinical specimens was then evaluated in a series of 108 well characterized breast cancer specimens, comparing HER2 immunofluorescence with PID to HER2 IHC with 3, 3′-diaminobenzidine (DAB)detection and HER2 ISH gene detection. Finally, we have investigated the potential clinical utility of PID quantification of HER2 protein in a cohort of HER2-positive breast cancers undergoing trastuzumab-based neoadjuvant therapy, looking for correlations with the pathologic response to treatment.

## Methods

### PID-nanoparticle preparation, staining and quantitation

The development and characterization of PID nanoparticles has been previously described [26]. The particles are comprised of perylene diimide assembly-conjugated nanoparticles that can be used for quantitative IHC detection of antibody labeled proteins in FFPE clinical samples or breast cancer cell lines. The PID nanoparticles undergo surface modification to attach an estimated 2460 streptavidin molecules per particle via PEG chains, which increases reactivity of the secondary antibody and promotes binding of the PID nanoparticles to the specified protein antigens. For reproducibility, accuracy and greater signal to noise ratio (S/N), each PID nanoparticle is created uniform in size (average size 149 nm), and has a fluorescent intensity that is 100-fold greater than other commercially available fluorescent nanoparticles and tissue auto-fluorescence.

Quantitative immunohistochemical detection of proteins using PID nanoparticles has been previously described [26]. Briefly, the samples are prepared by undergoing antigen retrieval and then immunostained with the primary antibody. The samples are then incubated with a biotinylated secondary antibody and treated with the pre-assembled PID nanoparticles (Fig. 1). The final sample is then stained with hematoxylin and mounted in mounting medium for viewing by bright field and fluorescent microscopy. The PID fluorescent signals are viewed using fluorescent microscopy independent of the optical viewing system. Three consecutive sections of the invasive tumor are used for PID analysis with five randomly selected fields from each section (approximately 1000 cells). The resulting fluorescent images are captured, processed and homogenized through a computer image-processing method which quantifies the number of PID nanoparticles per unit area (100μm^2^) and per cell which represents the protein expression levels [26], (Fig. 2).

### Analytic validation study

The methods and protocol for the analytic performance and validation of PID in HER2 testing has been previously described [26]. In the current study, six breast cancer cell lines (US Biomax, MD, USA) were used and are shown in Table 1. Briefly, cultured breast cancer cells (1.5 × 10^7^ cells) were fixed in 10% neutral buffered formalin mixed with alginic acid solution. The samples were embedded with alginate gel and treated with paraffin to prepare a paraffin block using a dedicated device (Retratome REM-700, Yamato Kohki, Saitama, Japan). The samples were cut into 4 μm thick sections and mounted on coated glass slides for analysis. For the Fluorescence-activated cell sorting (FACS) portion of the validation; FACS analysis method and protocol has been previously described [26]. Briefly, cultured breast cancer cells were fixed in 10% formalin, and the samples (2 × 10^6^ cells) were mixed with phosphate-buffered saline (PBS) containing 5% fetal bovine serum, 1 mM EDTA, and 0.1% NaN3. The samples were immunostained with 5 μg/mL primary antibody to HER2 (Anti-C-ErbB2/c-Neu(Ab-5) Mouse mAb(TA-1), Calbiochem, Merck Millipore, Tokyo, Japan) at 4 °C for 30 min. After a PBS wash, the samples were incubated with 1 μg/mL secondary antibody (Anti-Mouse IgG-Alexa Fluor488, Abcam, Tokyo, Japan) at 4 °C for 30 min. After another PBS wash, the samples were mixed with dedicated buffer for FACS and subjected to FACS measurements (MACSQuant Analyzer, Miltenyi Biotec, Bergisch Gladbach, Germany). We measured the fluorescence intensity per cell of 20,000 cells by FACS and calculated the mean value. Fluorescence-labeled beads (QIFIKIT Agilent Technologies, Santa Clara, CA) were also measured for calibration. QIFIKIT is used to determine the density of antibody-binding antigen per cell using FACS.

**Table 1:** Analytic validation study of cell lines

Cell Lines	Description	ER/PR/HER2 Status	Average PID score/ Average ST.DEV.
MCF7	Human epithelial breast mammary gland cells, positive for adenocarcinoma	+/+/−	11.5/0.6
T47D	Human epithelial breast mammary gland cells, positive for ductal carcinoma	+/+/−	40.6/2.9
HTB21	Human epithelial breast mammary gland cells, positive for adenocarcinoma	+/+/−	115.2/6.5
CRL1500	Human epithelial breast mammary gland cells, positive for ductal carcinoma	+/+/+	299.5/15.5
CRL1897	Human epithelial breast mammary gland cells, positive for ductal carcinoma	−/−/+	859.3/18.1
KPL4	Human epithelial breast mammary gland cells, positive for ductal carcinoma	−/−/++	1258.5/52.6

ER: estrogen receptor status, PR: progesterone receptor status, St.Dev.: standard deviation, (+): positive, (−): negative, (++): highly positive

### Method comparison study

#### Archival FFPE tissue block selection for study

The archives from the pathology department at the University of Rochester Medical Center were searched and 108 well characterized breast cancer cases were randomly selected that covered a range of HER2 results based on conventional IHC and ISH testing. Group-1 contained cases that were HER2 negative by IHC and ISH non-amplified; Group-2 contained cases that were HER2 positive by IHC and ISH amplified; Group-3 contained cases that were HER2 equivocal by IHC and ISH amplified and Group-4 contained cases that were HER2 equivocal by IHC and ISH equivocal. The HER2 PID score/cell and PID score/ROI100μm^2^ was measured as described above for each of these cases and compared against HER2 IHC manual interpretation and HER2 ISH results for the same cases.

#### IHC method

In the current study, HER2 protein expression in clinical samples were evaluated using HercepTest (anti-HER2 polyclonal antibody, Agilent Technologies, Santa Clara, CA) according to the protocol described in the manufacturer’s guide accompanying the testing kit. The IHC-DAB methodology and utility have also been previously descried [27]. Manual interpretation of IHC-DAB stained slides were done by a URMC pathologist and scored as (0–1+) IHC negative, (2+) IHC equivocal, or (3+) IHC positive.

#### Method for ISH hybridization and interpretation

The HER2 gene status was evaluated using the *HER2* IQISH pharmDX ™ (DAKO Denmark) according to the protocol described in the manufacture’s guide accompanying the testing kit. The ISH methodology and interpretation has also been previously described [27]. ISH results were reviewed by a pathologist at URMC and scored based on the 2013 ASCO HER2 guideline update [15].

### Neoadjuvant study (PID scores and correlation with pathologic response to therapy)

Thirty-four cases of HER2-positive breast cancer (determine by IHC and/or ISH from initial core needle biopsy), who had undergone neoadjuvant chemotherapy plus HER2-targeted therapy were selected for this portion of the study. Clinical pathologic variables including the post-treatment residual cancer burden (RCB) score in the resection specimens, nuclear grade and ER/PR status were recorded [28, 29]. Quantitative assessment of HER2 protein was determined using PID testing on the pre-treatment biopsy and correlated with the pathologic response to neoadjuvant therapy.

### Statistical analysis

To assess the correlations between PID scoring and other patient demographics (HER2 IHC, ISH, etc.), box and whisker plots were created to show the visualization of data. The box and whisker pots were created by separating the numerically ordered data into four quartile groups that included a lower quartile, containing the data below the 25th percentile, bounded by the lower whisker limit, an interquartile group containing the data between the 25th and 75th percentiles (including the lower interquartile range limit [Q1]), median quartile marking the mid-point of the data, and upper interquartile range limit [Q3]), and an upper quartile, containing the data above the 75th percentile, bounded by the upper whisker limit. Whisker limits and outliers (data falling outside the whisker limits) were determined with the upper whisker limit equal to [*Q3* + 1.5(*Q3* – *Q1*)], and the lower whisker limit equal to [*Q1*–1.5(*Q3* – *Q1*)]. Outliers are designated with a black circle outside the whisker limit. Mean values were also included in the boxplots, represented by the black diamonds, to further demonstrate statistical significance of our data. The F-test was used to test for homogeneity of variances. Parametric testing included coefficient of variation testing, paired t-testing, and ANOVA testing. Non-parametric testing included Mann-Whitney testing (Steel-Dwass method was used to determine significant differences between individual comparisons), and Kruskal-Wallis testing. Statistical calculations, figures and tables were created using Microsoft Excel (version Microsoft Excel Office 2010 version 14.0.7183.5000) and IBM SPSS software (version IBM SPSS 2017 version 23). For all results, a *p*-value of < 0.05 was considered statistically significant.

## Results

### Analytic validation study

**Fig. 3:**
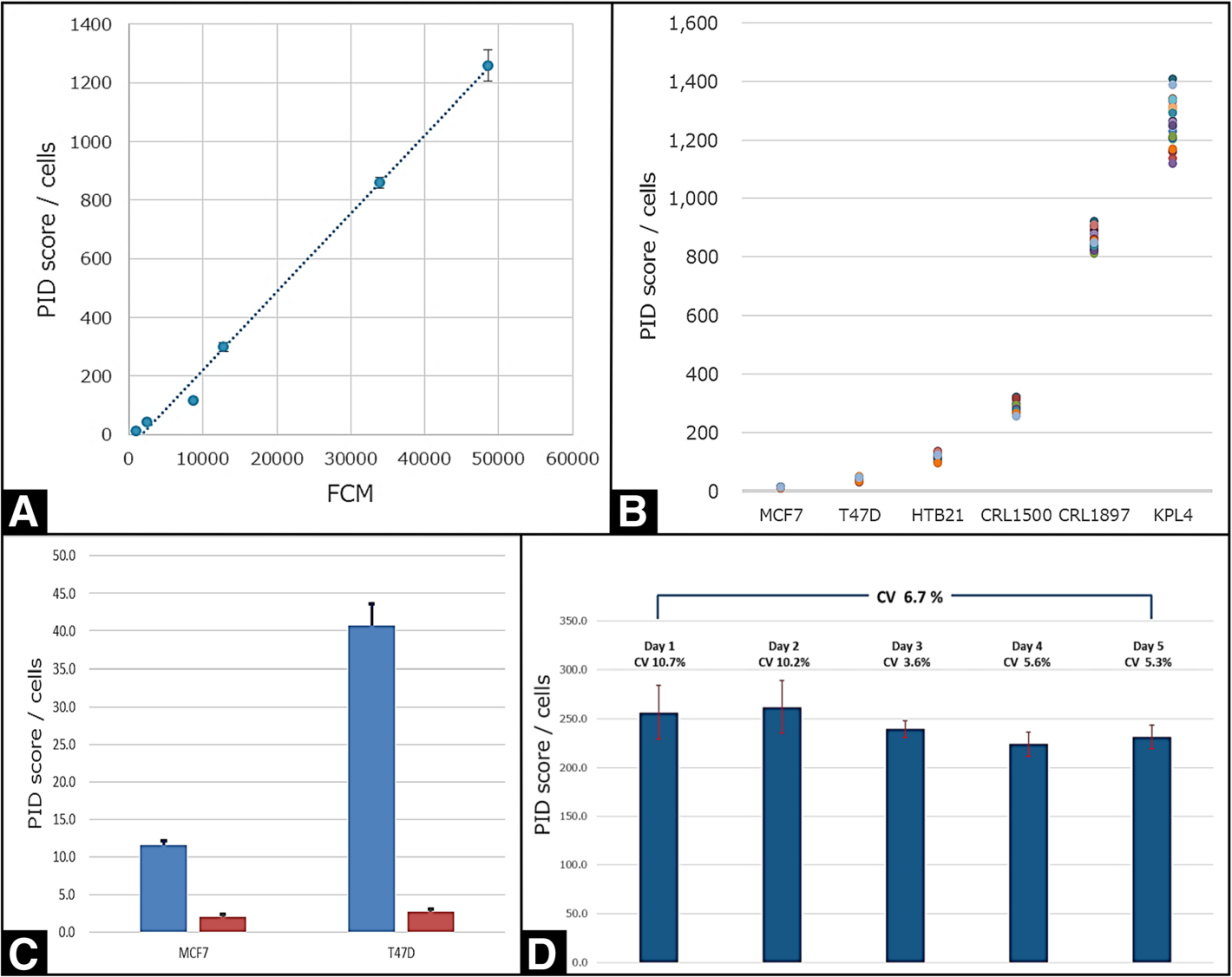
**a** HER2 expression was measured using FACS and PID in six types of cell lines. The test was conducted as 5 independent tests. Results of FACS and PID (per cell) were analyzed using Pearson correlation coefficient. Pearson correlation coefficient was 0.99. **b** HER2 expression was measured using PID in six types of cell lines. The test was conducted as 5 independent tests. Each experiment consisted of 5 slides. Blank experiment consisted of 3 slides (Blank = without primary antibody). A total of 25 slides were stained in each cell lines and 15 slides were stained in blank test. The separation between the each cell lines were tested by multiple comparison using Kruskal-Wallis test and Student’s t-test in each other. PID score of all 6 cell lines were statistical significantly different. **c** Analytical sensitivity test using HER2-expressing cell lines. PID score compared with blank test that was stained without primary antibody. In MCF7 cell line, the PID score / cells was 5.8 times that of the blank test and in T47D cell line, the PID score / cells was 15.0 times that of the blank test. **d** Schematic for reproducibility and robustness test on a single cell line. Twelve cut sections from one FFPE cell block were cut on 5 different days. Nine slides were tested with PID/IHC; three slides were tested without primary antibody (Blank) by four different technicians. A coefficient of variation of 6.7% for all the 5 days was obtained. Within-run variation ranged from 3.6 to 10.7%

In the current study, looking at the analytic validation, we demonstrated a significant level of accuracy for PID testing by comparing HER2 PID quantitation in cell pellets of six well categorized HER2 breast cancer cell lines to an established reference method (FACS analysis) in the same six cell lines (Fig. 3a., R^2^ = 0.99). PID precision was then demonstrated by conducting 5 independent tests on each of the six cell lines, each experiment consisting of 5 slides, and including a blank experiment consisting of 3 slides (Blank = without primary antibody). A total of 25 slides were stained in each cell line and 15 slides were stained in the blank test. The separation between each cell line was tested by multiple comparisons using the Steel-Dwass test and Kruskal-Wallis test. The PID score averages of all 6 cell lines were statistically significantly different (Fig. 3b.). Sensitivity was demonstrated by calculating a signal to noise (S/N) ratio using the criterion for detection capability in clinical laboratories proposed by CLSI EP17-A2 [30], which we adopted for PID to include the limit of detection requirement of (S/N) ratio above 3 and limit of quantification (S/N) ratio above 10. In this study, PID HER2 expression level of the MCF7 cell line was the limit of detection and the T47D cell line was the limit of quantification and, although the MCF 7 and T47D are defined as a score of 0 in the IHC-DAB method, it fell within the detection capacity of the PID method (Fig. 3c.). Expansion of capacity through digitization of images has also been confirmed in MDA-MB-231, which is also defined as an IHC score of 0 [31]. Lastly, robustness and reproducibility were then demonstrated by having 12 sections cut from one FFPE cell block on 5 different days and then stained by four different technicians. Within-run variation ranged from 3.6 to 10.7% and a coefficient of variation of 6.7% for all 5 days was obtained (Fig. 3d.)

### Method comparison study (HER2 breast cancer concordance)

**Fig. 4:**
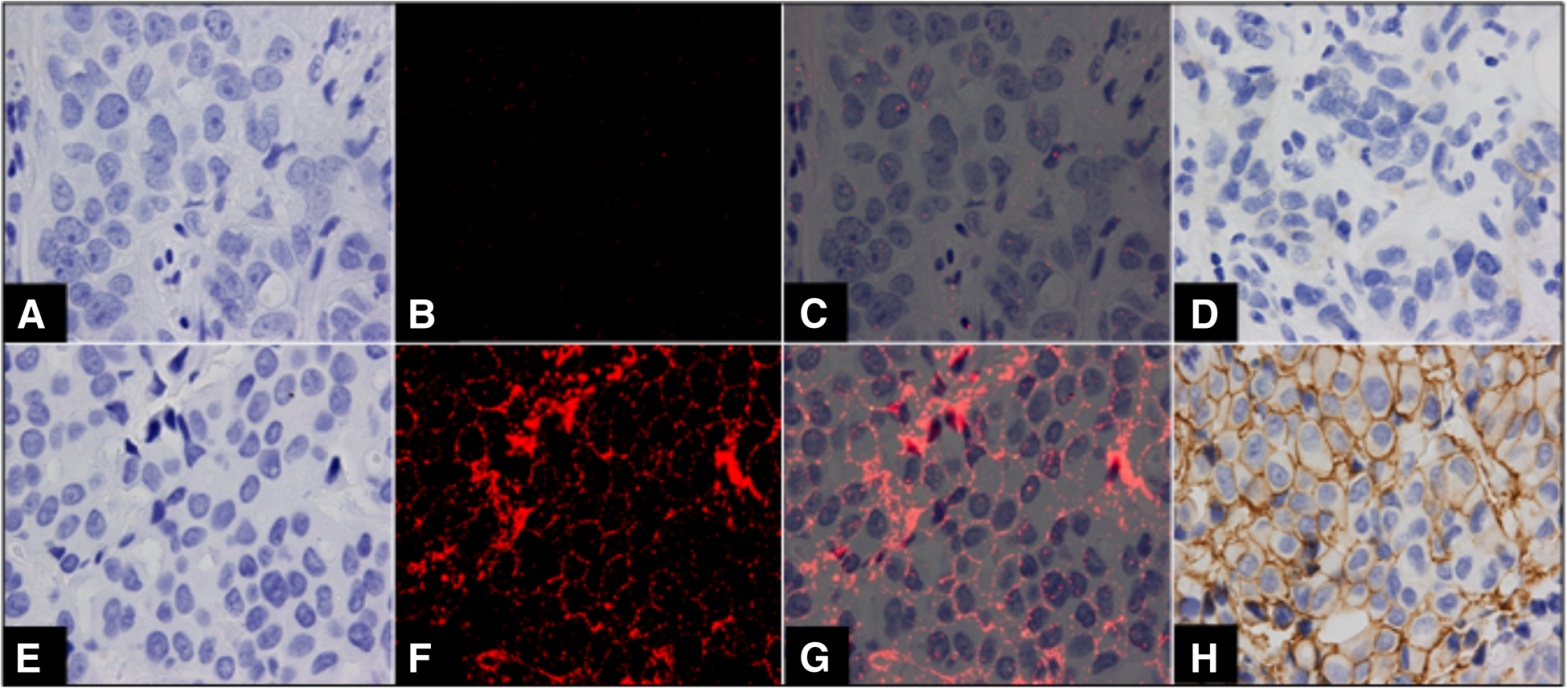
Examples of HER2 testing images evaluated with PID and IHC. Images **a**-**d** is a classic negative example and images **e**-**h** is a classic amplified example. Images A and E are of bright field images; images B and F are of florescent PID images; images C and G are an overlay of fluorescent PID and the bright field images; images D and H are of HER-2 IHC-DAB

Areas of invasive tumor were marked on a consecutive H&E stained slide to help target areas for PID analysis. The PID stained tissue sections were counterstained with Hematoxylin, which enables simultaneous imaging and evaluation of both morphology by bright field examination and PID labeling by fluorescence in the same tissue section (Fig. 4). The PID score/cell and PID/ROI100μm^2^ were quantitated utilizing the image-processing method described above, blinded to the HER2 IHC and ISH results. The quantitative results for PID/cell and PID/ROI100μm^2^ were then compared with HER2 IHC manual interpretation and HER2 ISH analysis. The results for the methods comparison study are summarized in Table 2.

**Table 2:** Methods comparison study data summary

Parameter	n (%)	PID/Cell (Mean, Median, St.Dev.)	PID/ROI100μm^2^ (Mean, Median, St.Dev.)
N Total	108		
IHC			
0	13 (12.0)	(3.5, 3.4, 1.6)	(2.4, 2.4, 1.2)
1+	16 (14.8)	(5.3, 4.6, 3.4)	(3.8, 2.7, 3.6)
2+	56 (51.9)	(31.9, 11.4, 56.5)	(16.3, 7.3, 24.1)
3+	23 (21.3)	(221.5, 204.4, 144.2)	(110.9, 125.6, 58.9)
FISH Ratio			
< 2	59 (54.6)	(7.9, 5.2, 7.3)	(5.4, 3.5, 5.9)
≥ 2	49 (45.4)	(133.6, 74.8, 141.6)	(66.1, 39.8, 63.4)
HER2 Copy #			
< 4	30 (27.8)	(6.6, 4.3, 7.4)	(5.0, 2.6, 7.0)
≥ 4, < 6	30 (27.8)	(8.8, 6.3, 7.1)	(6.1, 4.7, 5.0)
≥ 6	48 (44.4)	(136.5, 81.1, 141.7)	(67.1, 40.3, 63.6)
FISH Results			
Pos	56 (51.9)	(118.5, 57.1, 138.4)	(58.5, 23.3, 62.6)
Eq	23 (21.3)	(8.5, 6.2, 6.5)	(5.9, 4.7, 4.9)
Neg	29 (26.9)	(6.3, 4.0, 7.3)	(4.8, 2.6, 7.0)
IHC/FISH Result			
Neg/Neg	26 (24.1)	(4.4, 3.8, 3.0)	(3.1, 2.5, 3.0)
Eq/Eq	22 (20.4)	(8.7, 7.0, 6.5)	(6.1, 4.8, 4.8)
Eq/Pos	32 (29.6)	(48.9, 20.8, 69.9)	(23.6, 10.8, 29.5)
Pos/Pos	22 (20.4)	(229.9, 207.2, 141.9)	(114.3, 127.3, 57.9)
Neg/Pos	2 (1.9)	(5.5, 5.5, N/A)	(3.9, 3.9, N/A)
Neg/Eq	1 (0.9)	(4.7, 4.7, N/A)	(2.8, 2.8, N/A)
Eq/Neg	2 (1.9)	(15.5, 15.5, N/A)	(11.4, 11.4, N/A)
Pos/Neg	1 (0.9)	(37.6, 37.6, N/A)	(35.2, 35.2, N/A)

N: total sample size, n: subset sample size, (%): percentage of subset to total sample size, *IHC* immunohistochemistry, *FISH* fluorescent in situ hybridization, *neg* negative, *pos* positive, *eq* equivocal, *st.dev* standard deviation

### Comparison with HER2 IHC manual reads and ISH analysis versus PID

**Fig. 5:**
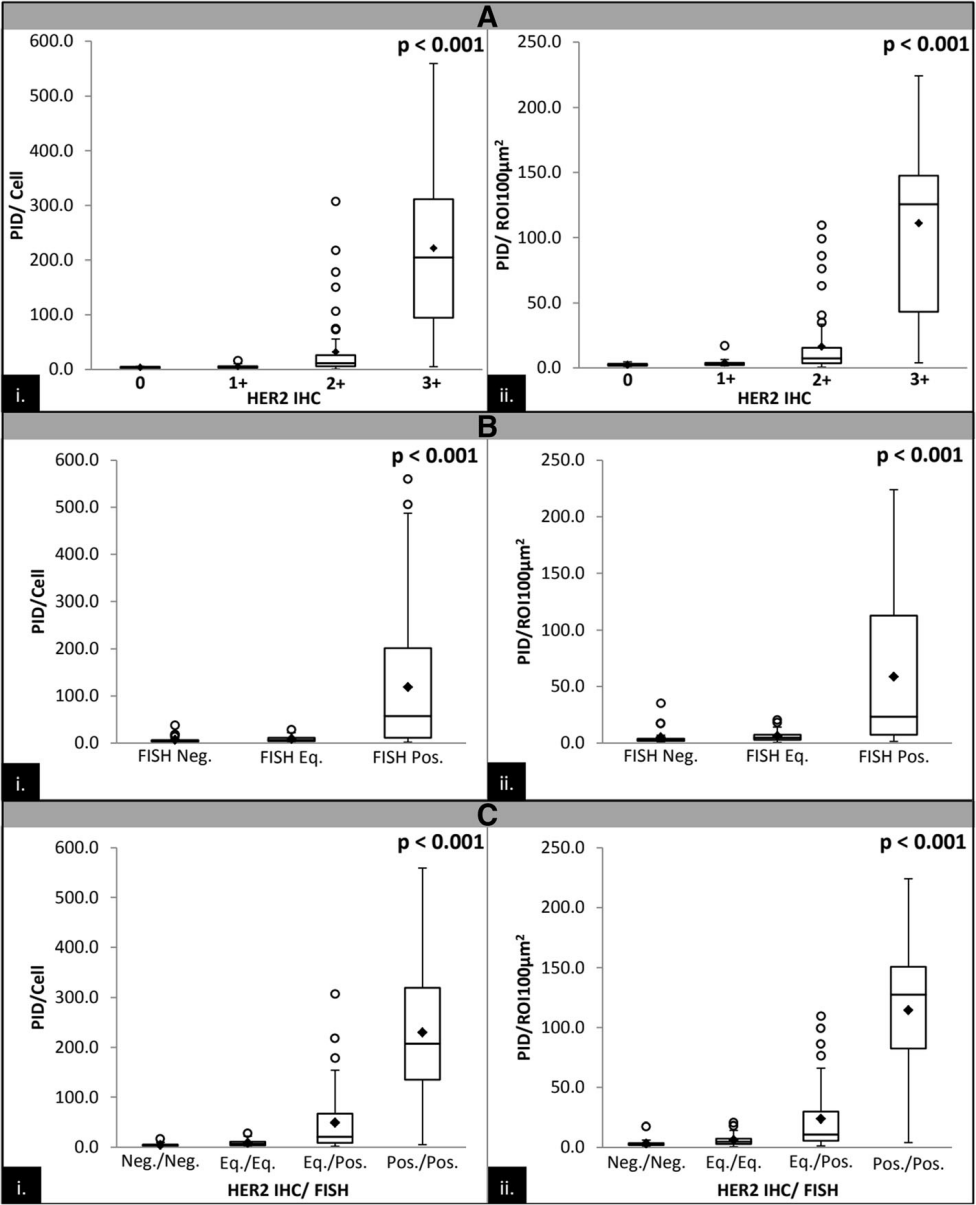
**a** Boxplot correlation between HER2 IHC score and PID from the method comparison study. Boxplot A is PID/cell vs. HER2 IHC 0, 1+, 2+, and 3+; boxplot B is PID/ROI100μm2 vs. HER2 IHC 0, 1+, 2+, and 3+. The black diamonds represent the mean PID score for each HER2 IHC score. The black circles represent the PID score outliers in each HER2 IHC score that are less than or greater than 1.5 times the lower and upper interquartile range limits, respectively. Both boxplots produced a *p* value < 0.001 from a non-parametric Kruskal-Wallis test. **b** Boxplot correlation between HER2 ISH categories (negative, equivocal and positive) and PID from the method comparison study. Boxplot A is PID/cell vs. HER2 ISH and boxplot B is PID/ROI100μm2 vs. HER2 ISH. The black diamonds represent the mean PID score for each HER2 ISH category. The black circles represent the PID score outliers in each HER2 ISH category that are less than or greater than 1.5 times the lower and upper interquartile range limits, respectively. Both boxplots produced a p value < 0.001 from a non-parametric Kruskal-Wallis test. **c** Boxplot correlation between HER2 IHC/ISH analysis (negative/negative, equivocal/equivocal, equivocal/positive and positive/positive) and PID from the method comparison study. Boxplot A is PID/cell vs. HER2 IHC/ISH and boxplot B is PID/ROI100μm2 vs. HER2 IHC/ISH. The black diamonds represent the mean PID score for each HER2 IHC/ISH category. The black circles represent the PID score outliers in each HER2 IHC/ISH category that are less than or greater than 1.5 times the lower and upper interquartile range limits, respectively. Both boxplots produced a *p* value < 0.001 from a non-parametric Kruskal-Wallis test

The PID/cell and PID/ROI100μm^2^ showed a strong and statistically significant correlation with HER2 IHC, HER2 ISH ratio, HER2 copy#, and HER2 concordant categories (Table 2, Fig. 5). The median PID/cell was 3.4 (SD 1.6) for cases scored as IHC (0), 4.6 (SD 3.4) for cases scored as IHC (1+), 11.4 (SD 56.5) for cases scored as IHC (2+) and 204.4 (SD 144.2) for cases scored as IHC (3+) (*P* < 0.001, Fig. 5a.i.). The PID/cell and PID/ROI100μm^2^ were also correlated with HER2 ISH results, but the correlation was not as strong as with IHC. The median PID/cell was 4.0 (SD 7.2) for ISH non-amplified cases, 6.2 (SD 6.4) for ISH equivocal cases and 57.1 (SD 137.1) for ISH amplified cases (P < 0.001, Fig. 5b.i.). Similarly, the PID/cell and PID/ROI100μm^2^ showed a correlation when compared with different HER2 categories. The median PID/cell was 3.8 (SD 3.0) for IHC and ISH negative cases, 7.0 (SD 6.5) for IHC and ISH equivocal cases, 20.8 (SD 69.9) for IHC equivocal and ISH positive cases and 207.2 (SD 141) for IHC and ISH positive cases (*P* < 0.001, Fig. 5c.i.).

### Correlation with pathologic response to neoadjuvant chemotherapy

**Fig. 6:**
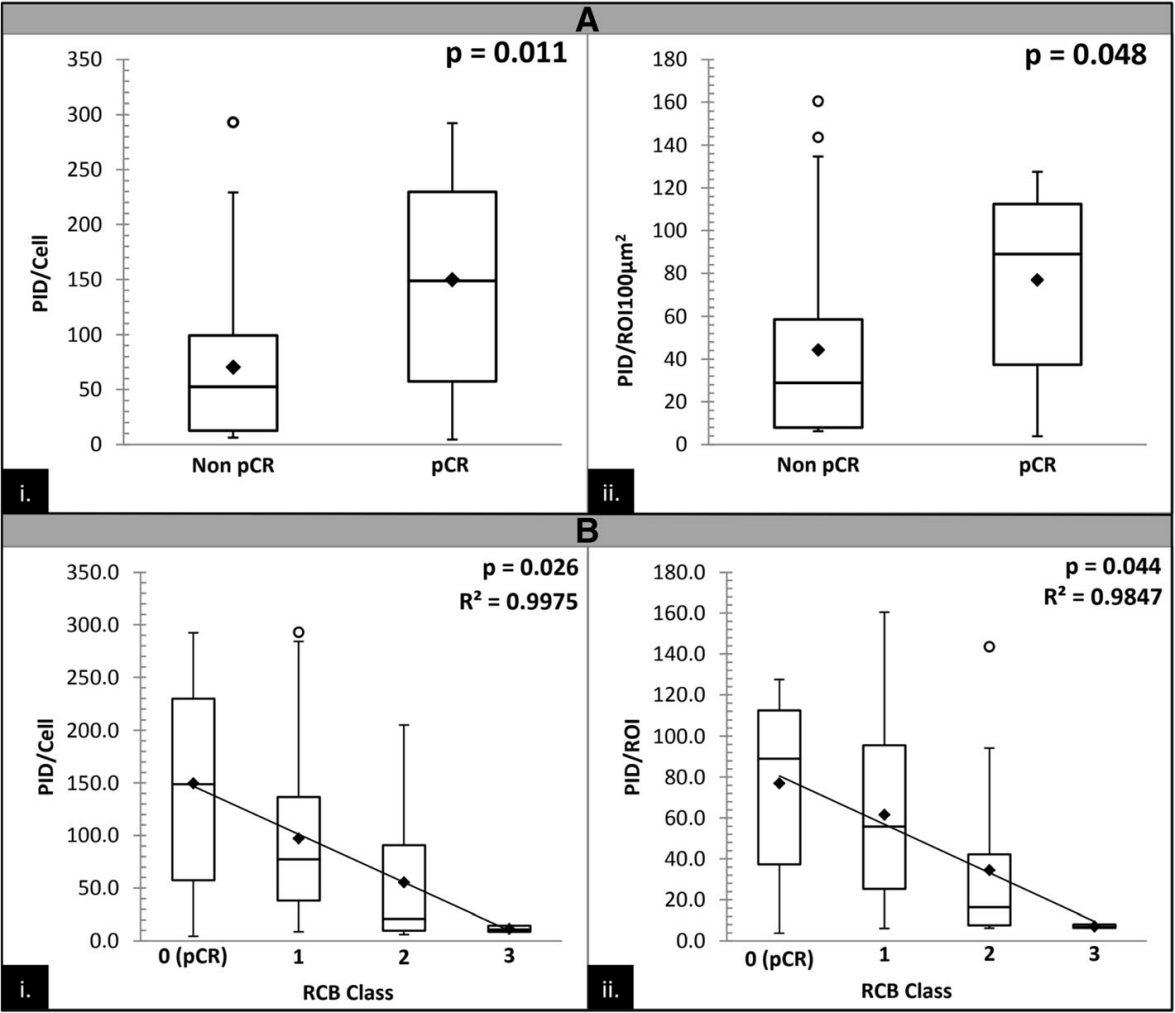
**a** Boxplot correlation between pathologic response (pathologic complete response or non-complete pathologic response) and PID score from the neoadjuvant study. Boxplot A.i. is PID/cell vs. pathologic response and boxplot A.ii. is PID/ ROI100μm2 vs. pathologic response. The black diamonds represent the mean PID score for both categories. The black circles represent the PID score outliers in both categories that are less than or greater than 1.5 times the lower and upper interquartile range limits, respectively. Boxplot A.i. produced a p value = 0.011 and boxplot A.ii. produced a p value = 0.048 from a paired t-test. **b** Boxplot correlation between RCB class (0 (pCR), 1, 2 and 3) and PID from the neoadjuvant study. Boxplot B.i. is PID/cell vs. RCB class and boxplot B.ii. is PID/ROI100μm2 vs. RCB class. The black diamonds represent the mean PID score for each RCB class. The black circles represent the PID score outliers in each RCB class that are less than or greater than 1.5 times the lower and upper interquartile range limits, respectively. Boxplot B.i. produced a p value of 0.026 and an R^2^ correlation value of 0.9975 between the mean PID score of each RCB class; boxplot B.ii. produced a p value of 0.044 and an R^2^ correlation value of 0.9847 between the mean PID score of each RCB class

**Fig. 7:**
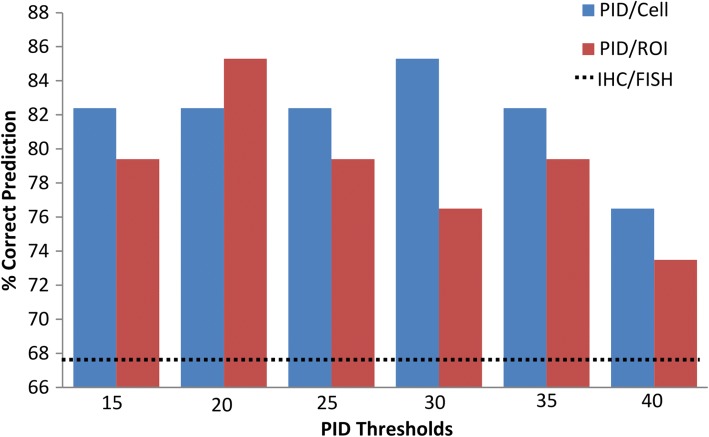
Bar graph comparing each experimental PID threshold [15, 20, 25, 30, 35, 40, and] from the neoadjuvant study to the correlating % of correct prediction to pathologic response. Briefly, if a specimen’s PID score fell above the experimental threshold then it would be predicted to have a good pathologic response (RCB 0 (pCR) or 1) and if the PID value fell below the experimental threshold then it was predicted to have a poor pathologic response (RCB 2 or 3). The best experimental thresholds from this set of data were found to be a PID/Cell of 30 and a PID/ROI100μm2 of 20 which both correctly predicted the pathologic response in 85.3% of cases. The black dotted line represents the % of cases that HER2 IHC and ISH analysis could correctly predict pathologic response using the guidelines from ASCO/CAP; which could only predict the correct pathologic response in 67.6% of cases

**Fig. 8:**
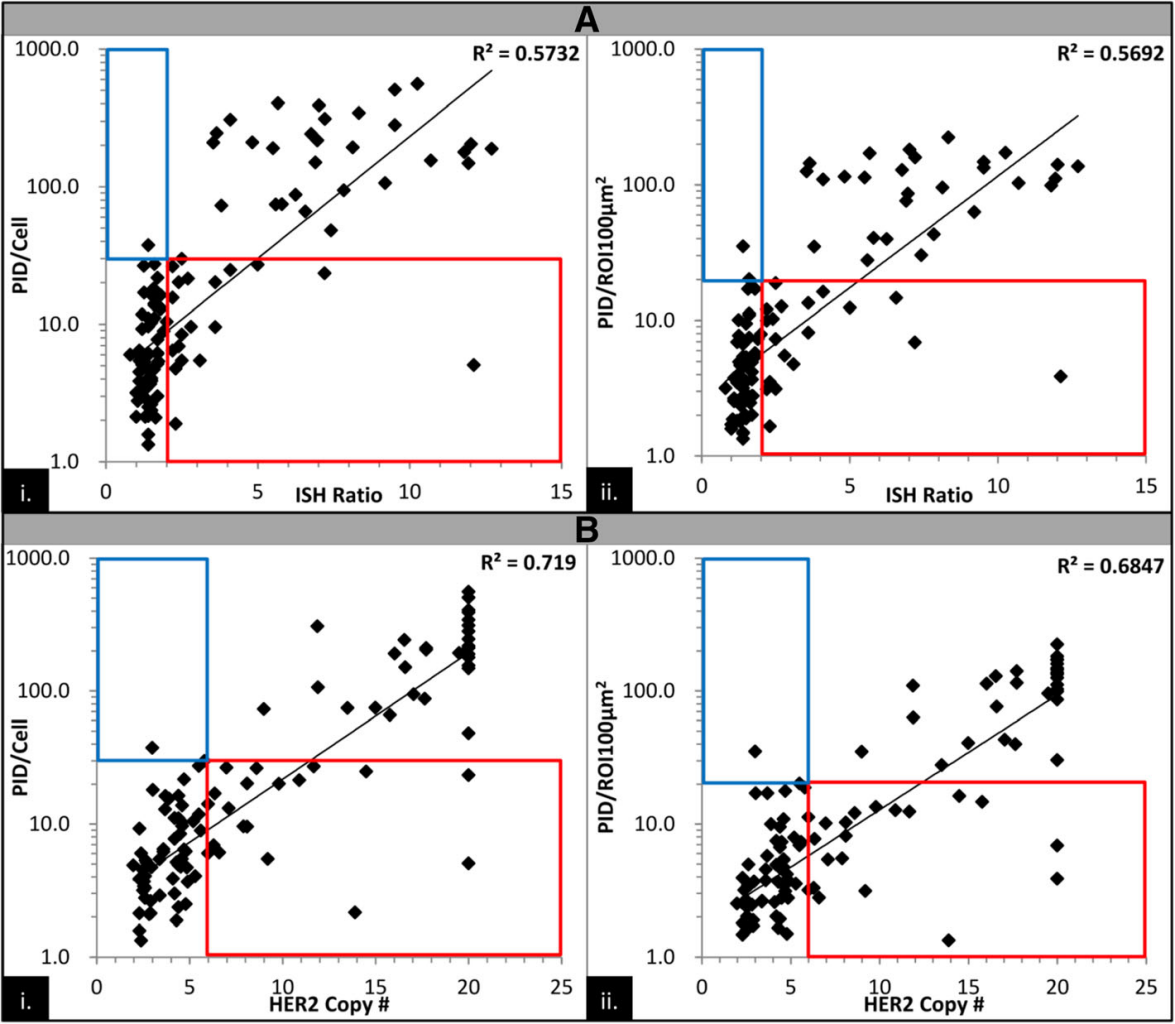
Scatter plot comparison of PID score to ISH data from the method comparison study. Graphs **A**.i. (R^2^ = 0.5732) and **A**.ii. (R^2^ = 0.5692) compare PID/cell and PID ROI100μm2 to ISH ratio, respectively. Graphs **B**.i. (R^2^ = 0.719) and **B**.ii. (R^2^ = 0.6847) compare PID/cell and PID ROI100μm2 to ISH HER2 copy number, respectively. The red box represents discordant results between PID analysis and ISH analysis utilizing the PID experimental thresholds from the neoadjuvant study and a ISH ratio of 2 or a HER2 copy number of 6 that resulted in a negative PID score but a positive ISH score. The blue box represents discordant cases that had a positive PID but a negative ISH result

As a pilot study, we sought to investigate the potential clinical utility of measuring HER2 protein receptors with PID testing in a cohort of 34 HER2-positive (defined by IHC and/or ISH analysis) breast cancer patients, who had undergone trastuzumab-based neoadjuvant therapy, to see if the quantitative measurement of HER2 protein receptors correlated with pathologic response to therapy. The pathologic response was scored using the residual cancer burden score (RCB), which has been shown to be highly correlative for predicting survival after neoadjuvant chemotherapy for all breast cancer phenotypes [9, 15]. The patient demographics and clinicopathological characteristic for the cohort are shown in Table 3. The correlation with pathologic response and PID scores are shown in Fig. 6. Patients with a pCR had a significantly higher mean (149.8, SD 77) and median (148.7, SD 89) PID score/cell (Fig. 6a.i., *p* = 0.011) and PID score/ROI100μm^2^ (Fig. 6a.ii., *p* = 0.048) compared with patients who did not achieve a pCR. To further investigate the relationship of PID and pathologic response, we explored the correlation of PID score to each RCB class in a box and whisker plot (Fig. 6b.). The figure shows a linear relationship that is statistically significant for both PID/cell (Fig. 6b.i., *p* = 0.026; R^2^ = 0.9975) and PID/ROI100μm^2^ (Fig. 6b.ii., *p* = 0.044; R^2^ = 0.9847), which shows that PID is highly correlated suggesting that the PID score might predict pathologic response. Utilizing this information, experimental PID thresholds of 15, 20, 25, 30, 35, and 40 were proposed; a score greater than the threshold was considered PID positive and a score less than the threshold was considered PID negative in terms of predicting pathologic response to neoadjuvant therapy. For each proposed experimental threshold, any PID score over the proposed threshold value (PID positive) was considered a “positive-predictor” and therefore would correspond to a good pathologic response (RCB class 0 or 1) to targeted therapy and thus; any PID score lower than the proposed threshold (PID negative) was considered a “negative-predictor” and therefore would correspond to a poor pathologic response (RCB class 2 or 3) to targeted therapy. We then calculated the percentage of patients that PID correctly predicted the response to therapy based on these thresholds (Fig. 7). All 34 patients in the neoadjuvant study were considered IHC and/or ISH positive based on the ASCO/CAP standardized recommendations [15], and thus, all patients would have been predicted to have a good response to therapy. However, 11 out of the 34 patients had a poor pathologic response (RCB class 2 or 3) to therapy which shows that current methodologies could only correctly predict response in 23 out of 34 (67.6%) patients. Both a PID/Cell threshold of 30 and a PID/ROI100μm^2^ threshold of 20 could correctly differentiate and predict which of these patients would have a good response (RCB class 0 or 1) or a poor response (RCB class 2 or 3) to therapy in 29 out of 34 (85.3%) patients. Figure 8 shows a graphic, scatter plot comparison of the ISH ratio and the average HER2 copy number versus the PID/cell and PID/ROI100μm^2^ from the methods comparison study, and shows a good correlation (R^2^ = 0.5732 to R^2^ = 0.719). If we apply the positive predictive thresholds proposed in the neoadjuvant study, in Figs. 8a.i. and 8A.ii., the RED box indicates the range of data that has a ISH ratio greater than 2 but for Fig. 8a.i., PID/cell *less than* 30 and Fig. 8a.ii., PID/ROI100μm^2^ *less than* 20 (best experimental thresholds from neoadjuvant pilot study above). These cases represent discordant cases (ISH amplified by ratio, PID low). The BLUE box indicates the range of data that includes ISH ratio less than 2 but Fig. 8a.i., a PID/Cell *greate*r *than* 30 or Fig. 8a.ii., PID/ROI100μm^2^ *greater than 20* (same thresholds); these cases are also discordant (ISH non-amplified, PID high). Cases not in the red or blue box are PID and ISH ratio concordant. For Figs. 8b.i. and 8B.ii., these plots show the HER2 Copy #, and the same approach was applied, with a cutoff range for a HER2 Copy # of *greater than* (red box) or *less than* (blue box) 6, correlated with a PID/cell (Fig. 8b.i.) *less than* (red box) or *greater than* (blue box) 30, respectively. PID/ROI100μm^2^ *less than* (red box) or *greater than* (blue box) 20, respectively was also utilized (Fig. 8 b.ii.).

**Table 3:** Neoadjuvant study, data demographics summary

Parameter	n (Average)	% (Range)
N Total	34	
Age	(54)	(23–83)
Diagnosis (DX)		
Invasive Ductal Carcinoma	33	97.1
Invasive Lobular Carcinoma	1	2.9
Nuclear Grade		
1	2	5.9
2	7	20.6
3	25	73.5
ER Allred Score		
0–2 (−)	11	32.4
3–8 (+)	23	67.6
PR Allred Score		
0–2 (−)	13	38.2
3–8 (+)	21	61.8
ER/PR Status		
ER+/PR+	21	61.8
ER+/PR-	2	5.9
ER-/PR+	0	0
ER-/PR-	11	32.4
HER2 IHC Score		
0	*1*	2.9
1+	0	0
2+	10	29.4
3+	23	67.6
Pathologic Response		
Partial/No Response	11	32.4
Near complete Response	11	32.4
Complete Response (pCR)	12	35.3
Residual Cancer Burden (RCB) Class		
0 (pCR)	12	35.3
1	11	32.4
2	8	23.5
3	3	8.8

N: total sample size, n: subset of total sample size, *ER* estrogen receptor, *PR* progesterone receptor, *IHC* immunohistochemistry, *pCR* pathologic complete response. *1 patient presented with an IHC score of 0 but was ISH amplified and received HER2 neoadjuvant therapy and was included in the cohort.*

## Discussion

The development of effective targeted therapy for breast cancer patients who over-express HER2 represents a major advancement in medical oncology and has arguably played a large role in heralding in a new era of companion diagnostics and predictive cancer biomarker guided therapeutic decision making [3–6]. The clinical assays currently used to assess the HER2 status in patients being considered for targeted therapy include IHC and ISH, which both have limitations; furthermore, the accuracy and reliability of these methods in routine clinical practice for patient selection remains a subject of debate. In the current study, we have used HER2 receptor expression in breast cancer as a model to explore the potential clinical utility of a novel detection technology using streptavidin coated Phosphor Integrated Dot fluorescent nanoparticles (PID) for the quantitative measurement of proteins of clinical interest in breast cancer cell lines and routine clinical samples. In a series of analytic performance studies using well characterized breast cancer cell lines, our PID method demonstrated sensitive, accurate, precise and robust quantitative measurements of the HER2 protein. We next compared HER2 PID measurements against conventional IHC and ISH analysis in 108 well characterized breast cancer samples that were randomly selected to cover a range of HER2 results. Similarly, in this methods comparison study, HER2 PID measurements showed a strong correlation with conventional IHC and ISH methods in this series of well characterized clinical breast cancer samples. Of note, IHC showed a better correlation with PID measurements compared with ISH and there was a range of PID values seen for cases that were considered IHC 3+ (median PID/cell 204.4, SD 144.4) and ISH amplified cases (median PID/cell 57.1, SD137.1).

ISH analysis, which measures average HER2 gene copy number, is a surrogate for HER2 receptor over-expression and relies on the assumption that the amount of DNA copy numbers detected will accurately reflect the amount of protein that is eventually translated in tumor cells [20]. This is likely an oversimplification, given the intricacy of the potential regulatory influences and cell processes involved in the expression of receptor tyrosine kinases such as HER2. While gene amplification is considered to be the primary mechanism of HER2 over-expression in breast cancer [31, 32], the biologic regulation of HER2 expression in tumor cells is complex and multifaceted. Gene amplification may not always correlate quantitatively with HER2 protein expression. In fact, in the methods comparison portion of the current study, we noted a broad range of HER2 protein receptor values measured by PID-scores for cases that showed HER2 gene amplification with a subset of cases that had discordance between the ISH and PID results (Fig. 8). Similarly, other studies have reported discordant results between gene amplification and quantitative HER2 protein expression where 13% of central ISH positive tumors showed discordant low levels of HER2 protein [33]. The ability to directly and accurately measure the protein levels with PID-nanoparticles may potentially circumvent the impact of transcriptional or translational regulatory events that may influence HER2 receptor expression and subsequent disease progression [34, 35].

Although accuracy and reproducibility of the PID assessment of HER2 was confirmed in the analytic validation and method comparison portions of this study, pre-analytical variables have the real potential to affect the quality of quantitative HER2 results. A previously published investigation of cold ischemic time on HER2 assay results showed that cold ischemia time up to 3 h has no deleterious effect on the detection of ERBB2 via in situ hybridization or IHC (Porter, et al., Mod Path 2013). This is certainly a potential limitation to any quantitative protein assay and clinical data is clearly needed to understand the relationship between quantitative HER2 protein expression measurements, pre-analytical variables and clinical outcomes in breast cancer patients treated with HER2-targeted therapy, especially in institutions that do not closely monitor pre-analytical variables.

As a pilot study, we sought to investigate the potential clinical utility of evaluating HER2-PID scores in the pretreatment biopsies from 34 HER2-positive carcinomas that had undergone neoadjuvant trastuzumab-based chemotherapy. Our goal was to assess correlations of HER2-PID scores with the pathologic response to treatment. The pathologic response to neoadjuvant chemotherapy has been shown to be prognostic and has been proposed as a surrogate endpoint for prediction of long term clinical benefit [29, 36]. Patients who achieved a pCR in our study had a significantly higher mean and median PID score/cell compared with patient who did not achieve a pCR (*p* = 0.011, Fig. 6a.). The mean and median PID scores also showed an inverse correlation with the RCB-class, which separates patients into groups based on the degree of the pathologic response seen after neoadjuvant therapy (ranging from zero or pCR to RCB class-3 representing a poor response, Fig. 6b.). Although limited by retrospective analysis, a small sample size, and lack of randomization or uniform therapeutic regimens, our findings suggest that quantitative HER2 expression levels may provide improved specificity in the selection of patients who are more likely to benefit from HER2-targeted therapy.

Conventional immunohistochemistry (IHC) with 3.3’diaminobenzidine chromogen detection is extensively used as a diagnostic tool in pathology for the detection of a wide range of proteins in FFPE clinical samples. Conventional IHC is a powerful technique, allowing the simultaneous evaluation of tissue morphology as well as the localization of specific proteins within different tissue compartments; however there are limitations in its application for predictive biomarkers in cancer. The chromogenic detection employed by IHC has a restricted dynamic range where the test is linear, limiting the ability to obtain accurate and quantitative results. The intensity of the chromogenic staining depends on the enzymatic activity of the detection system being employed and is significantly influenced by the reaction time, temperature and the substrate concentration, thus limiting the quantitative sensitivity of IHC [17, 31].

It is clear that, among breast cancers defined as “HER2-positive” by conventional testing methods, there is a wide range of variability in terms of the level of gene amplification and/or protein over-expression in tumor cells. The likelihood that a more quantitative analysis of HER2 protein expression could improve prediction of the response to HER2-targeted therapy has led to an exploration of methods that are more accurate and quantitative for protein expression levels. Numerous attempts have been made to develop such quantitative methodologies for the measurement of predictive biomarkers, such as HER2 in breast cancer samples. Jensen et al. reported on a novel amplification system (qIHC) that enables quantification of proteins directly in FFPE samples by counting dots [30] which showed the ability to measure HER2 protein accurately and over a large dynamic range in cell lines and human breast cancer tissues. This study showed a good correlation between their qIHC method and standard HER2 IHC measurements in 44 breast cancer clinical specimens; however, no data on clinical utility was reported. Miyashita et al. has developed an IHC technique with quantum dot conjugated trastuzumab for measuring HER2 that correlated proportionally with HER2 gene copy number assessed by ISH (*R* = 0.83). Furthermore, the level of expression of HER2 measured by this method correlated with time to progression after trastuzumab therapy in 37 HER2 positive cancers (*R* = 0.69) [37]. Nuciforo et al. used selected reaction monitoring mass spectrometry (SRM-MS) to quantify HER2 protein levels in FFPE breast cancer samples [38]. They showed that the absolute HER2 levels measured by SRM-MS were significantly correlated with both IHC and ISH and that their quantitative measurement of HER2 was superior to IHC and ISH for predicting outcome after treatment with HER2-targeted therapy in the adjuvant and metastatic settings [38]. Cheng et al. used quantitative immunofluorescent measurements (AQUA technology) to assess HER2 protein in core biopsies from 27 HER2-positive breast cancers enrolled in a preoperative clinical trial using trastuzumab-based chemotherapy [39]. They showed a significant correlation between high levels of HER2 measurements (mean 10,251) and pCR compared with patients without a pCR (mean 4766, *p* = 0.0021). This finding is strikingly similar to what is being reported in the current study and suggests that the accurate measurement of HER2 protein may help determine the likelihood of response in the neoadjuvant setting. Similarly, many other studies investigating the response to neoadjuvant HER2 targeted therapy have shown that high HER2 levels [16, 40], *HER2/CEP17* ratio > 6 [41], high baseline tumor infiltrating lymphocytes [42] and HER2-enriched molecular subtype [43] are all associated with a significantly higher pCR rate. Each of these reported predictors of pathologic response to neoadjuvant therapy is likely to be associated with higher levels of HER2 receptor protein expression in tumor cells as well as possible immune mechanisms of action for HER2-targeted therapy [42].

The HERmark Breast Cancer Assay (Monogram Biosciences, South San Francisco CA) provides another method for precise quantitation of total HER2 protein expression and HER2 homodimers in FFPE breast cancer specimens [20]. When compared against centrally performed IHC and ISH results from 237 breast cancer cases, HERmark showed a 98% concordance with IHC positive and negative assay values [20]. For 94 IHC equivocal cases, 67 and 39% concordance results were seen between HERmark and ISH amplified and non-amplified results respectively. Yardley et al. has examined the prognostic significance of quantitative measurement of HER2 by HERmark in a cohort of 192 breast cancer cases enrolled in a multicenter collaborative biomarker study, most of whom received no HER2-targeted therapy [44]. While the HERmark assay results correlated well with routine HER2 testing, there was a range of levels of expression of HER2 protein measured by HERmark. When the HERmark and local test results were discordant (local testing negative, HERmark discordantly high or local testing positive, HERmark discordantly low) HERmark more accurately predicted overall survival. This result suggests that the quantitative measurement of HER2 protein may more accurately reflect the underlying tumor biology in cancers that are labeled as “HER2-positive” by routine testing in terms of prognosis. Lipton et al. has reported on the potential role for quantitative measurement of HER2 protein by HERmark for predicting outcome after treatment with trastuzumab in metastatic breast cancer patients. In this study of 98 patients who had central ISH and HER2 total expression results, Cox proportional hazards multivariate regression identified HER2 total expression as an independent predictor of time to progression (HR = 0.29, *P* = 0.0015) and overall survival (HR = 0.19, *P* < 0.001). These authors concluded that a subset of patients with HER2 gene amplification by ISH express low levels of HER2 protein and have a reduced response to HER2-targeted therapy, similar to ISH-negative cases [33].

## Conclusions

PID nanoparticles demonstrate great potential for the quantitative measurements of proteins of clinical interest in routine clinical samples with morphologic confirmation of the tissue being studied. Using HER2 expression in breast cancer as a model, we have shown that PID measurements demonstrated sensitive, accurate and precise quantitation of HER2 protein in six breast cancer cell lines, is highly correlated with conventional HER2 testing methodology in clinical samples, and may be useful for predicting pathologic response to trastuzumab-based neoadjuvant therapy. Several major limitations are present in the current study, which could potentially confound these analyses, including 1) the small sample size that was used to evaluate the correlation between the PID scores and the pathologic response to neoadjuvant therapy, 2) the retrospective nature of the study, and 3) the non-uniformity of the treatment regimens received. Thus our findings should be considered preliminary and hypothesis-generating until they can be confirmed in additional larger, well-controlled neoadjuvant studies for patients with HER2-positive breast cancer. That being said, our data suggests that the quantitative measurement of HER2 protein levels, where the drug is targeted, may offer an advantage over conventional testing in the selection of patients who will be the most likely to benefit from HER2-targeted therapy, which is a conclusion that is supported by other reports in the literature. Further studies in a larger patient cohort undergoing neoadjuvant therapy are warranted.

## Abbreviations

(S/N): Signal to noise
CEP17: Chromosome enumeration probe 17
DAB: Diaminobenzidine
ER: Estrogen receptor
FACS: Fluorescence-activated cell sorting
FFPE: Formalin fixed paraffin embedded
HER2: Human epidermal growth factor 2
IHC: Immunohistochemistry
ISH: In situ hybridization
PBS: Phosphate-buffered saline
pCR: Pathologic complete response
PID: Phosphor integrated dot fluorescent nanoparticles
PR: Progesterone receptor
qIHC: Quantitative immunohistochemistry
RCB: Residual cancer burden
ROI100μm^2^: Region of interest 100 μm squared
SD: Standard deviation
URMC: University of Rochester Medical Center
